# Multimodal classification of skin lesions based on lightweight gated low-rank hyper-fusion network

**DOI:** 10.3389/fdgth.2026.1947161

**Published:** 2026-10-08

**Authors:** Ke Wang, Tao Zhang, Xingqi Liu, Jiancun Zhou, Qian He, Jianxin He

**Affiliations:** 1 College of Information and Electronic Engineering, Hunan City University, Yiyang, China; 2 College of Mechanical and Electrical Engineering, Hunan City University, Yiyang, China

**Keywords:** explainable AI, gating mechanism, low-rank hypernetwork, multimodal fusion, skin lesion classification, Swin Transformer

## Abstract

Skin cancer imposes a substantial global public health burden, and early, non-invasive, and accurate lesion classification is a critical prerequisite for improving patients' long-term survival outcomes. In routine dermatological practice, multimodal integration of dermoscopic images and clinical metadata (including patient age, sex, and lesion anatomical location) has been widely adopted. Nevertheless, current state-of-the-art multimodal fusion frameworks still encounter a set of unresolved critical bottlenecks in real-world clinical deployment. These unaddressed limitations primarily include cross-modal negative transfer, overfitting under limited annotated sample regimes, insufficient inter-modal feature interaction, and the “black-box” nature of model decision-making, all of which collectively degrade the operational robustness and real-world clinical deployment feasibility of existing multimodal diagnostic frameworks. To systematically address these unmet challenges, this work presents a Gated Low-Rank Hyper-Fusion Network (GLHF-Net) for automated skin lesion classification. The proposed architecture leverages a lightweight Swin Transformer variant as its visual feature extraction backbone, built around a two-stage dynamic multimodal fusion pipeline. First, a Metadata-Driven Channel-wise Gating Mechanism (Meta-Gate) adaptively suppresses task-irrelevant channel-level noise in high-dimensional visual feature maps, mitigating cross-modality distribution discrepancy between tabular clinical metadata and latent image embeddings. Second, an embedded Low-Rank Hyper-Dynamic Fusion (LR-HDF) module executes constrained deep feature reconstruction under explicit low-rank regularization, effectively reducing empirical overfitting risk on small annotated dermatological datasets. Extensive validation experiments carried out on the public benchmark dataset HAM10000 demonstrate that the proposed GLHF-Net model achieves robust diagnostic accuracy with an AUC of 0.9200, a pAUC of 0.8405, a Sensitivity of 0.8935 and an NPV of 0.9682, and demonstrates highly competitive performance compared to both conventional feature concatenation methods and methods based on complex attention mechanisms. Grad-CAM visualization results confirm that the proposed model can accurately localize core lesion regions while effectively suppressing interference artifacts such as occluding hair, which provides crucial interpretable evidence for the clinical reliability of the model. With strong anti-overfitting ability and high inference efficiency (121.1 FPS on GPU and 20.35 FPS on CPU), this framework provides a reliable lightweight solution for the deployment of intelligent skin cancer screening in clinical scenarios.

## 1 Introduction

Skin cancer represents an escalating global public health burden, with incidence rates showing a sustained upward trend worldwide and a marked shift toward younger affected populations. Among all skin cancer subtypes, melanoma carries the highest clinical risk due to its strong malignant potential and rapid disease progression. Notably, the clinical prognosis of melanoma patients is highly sensitive to the timeliness of diagnosis and intervention. Clinical evidence indicates that the 5-year survival rate for patients with early-stage melanoma receiving standardized treatment exceeds 95%; in contrast, for advanced-stage disease, this rate drops sharply to below 20%. Early diagnosis and standardized treatment remain the core strategies for improving patient prognosis and reducing disease-related mortality (1, 2). Accordingly, developing an accurate, non-invasive, and efficient technology for benign–malignant classification of skin lesions holds great practical significance for reducing individual patient burden and improving clinical diagnostic and treatment capacity for skin cancer.

Dermoscopic imaging has become a cornerstone of modern dermatological practice, recognized for its non-invasive nature, high resolution, and operational convenience. As an essential tool, it is widely applied to primary diagnosis, long-term monitoring, and population-level screening of skin diseases, providing high-quality visual data to support artificial intelligence (AI)-assisted diagnostic research (3, 4). In recent years, deep learning has advanced rapidly in medical image analysis. Convolutional Neural Networks (CNNs) represented by ResNet, DenseNet, and EfficientNet can automatically extract shallow visual features such as texture, color, boundary and morphology of skin lesions through multi-layer convolution and pooling operations. Their classification performance is significantly superior to traditional manual feature extraction and machine learning methods, thus they have been widely applied to intelligent diagnosis tasks of skin lesions (5–7). However, the inherent limitation of the local receptive field in CNNs makes it difficult to model the global spatial structure, long-range feature dependencies, and irregular morphology of skin lesions. In complex scenarios—including small lesions, low-contrast lesions, and images with strong background interference—CNNs are prone to missed and false detections, ultimately compromising diagnostic robustness.

In recent years, the Transformer architecture has effectively addressed the aforementioned limitations of CNNs. As a representative model for the successful application of Transformers in computer vision, Swin Transformer performs hierarchical feature extraction and global context modeling through a shifted window-based multi-head self-attention mechanism (8) while maintaining linear computational complexity. Owing to its superior feature representation and computational efficiency, Swin Transformer has become the mainstream backbone network for dermoscopic image analysis (9, 10). Although existing Swin Transformer-based single-modal diagnostic models achieve superior performance, they rely solely on visual lesion information, completely neglecting patient prior information that plays a critical role in clinical diagnosis and treatment.

Extensive clinical practice has confirmed that dermatologists’ diagnostic decision-making follows a comprehensive paradigm integrating visual lesion features with patient clinical information. Structured metadata—such as patient age, gender, and lesion anatomical location—hold irreplaceable diagnostic value in identifying benign vs. malignant skin lesions, determining subtypes, and stratifying malignancy risk (11, 12). Reasonable fusion of multimodal information from dermoscopic images and clinical metadata can compensate for the feature limitations of single-modal models, optimize decision-making logic, substantially improve diagnostic accuracy and robustness, and align with actual clinical workflows (13, 14). Therefore, constructing a multimodal diagnostic model driven by image–metadata collaboration represents a critical research direction for overcoming the performance bottleneck of single-modal skin lesion classification.

However, multimodal skin lesion diagnosis faces challenges including data heterogeneity, limited samples, and complex noise. Existing multimodal fusion methods have four core problems that seriously restrict the clinical application of models:

1. Prominent cross-modal negative transfer. Traditional early fusion methods directly concatenate high-dimensional image features with low-dimensional clinical metadata. The substantial dimensional discrepancy causes clinical prior knowledge to be overwhelmed by visual features, thereby failing to provide auxiliary diagnostic value and potentially introducing heterogeneous noise. This phenomenon leads to cross-modal negative transfer and degraded model performance (15, 16).
2. Significant risk of small-sample overfitting. Deep fusion models based on cross-attention and co-attention have a large number of parameters and high model capacity. However, skin lesion clinical labeled samples are scarce and the data distribution is uneven, leading to the model being extremely prone to overfitting on the training set and greatly reduced generalization ability (15, 17).
3. Excessively high deployment cost of dynamic fusion. Traditional hypernetworks achieve adaptive fusion by generating full-rank weight matrices. Both parameter counts and computational complexity scale quadratically with feature dimension, incurring substantial computational overhead and slow inference speeds that impede clinical real-time screening deployment (18, 19).
4. Insufficient clinical credibility in decision-making. Most existing multimodal fusion models operate as black-box systems that cannot intuitively display decision rationales or attention regions. Their diagnostic logic often diverges from clinical norms, thereby failing to gain physician trust or achieve clinical adoption (20, 21).

To address these challenges, we propose a lightweight Gated Low-Rank Hyper-Fusion Network (GLHF-Net) built upon the Swin Transformer architecture. The principal contributions are as follows:

1. To mitigate cross-modal negative transfer, we propose a Metadata-Driven Channel-wise Gating Mechanism (Meta-Gate). Prior to feature fusion, clinical metadata generates a channel attention mask that adaptively filters and purifies visual features, thereby effectively inhibiting cross-modal negative transfer induced by modal heterogeneity.
2. To alleviate small-sample overfitting, we design a Low-Rank Hyper-Dynamic Fusion (LR-HDF) module. By imposing rank constraints, the full-rank weight matrix of the hypernetwork is decomposed into the product of low-rank matrices, reducing parameter counts and effectively mitigating overfitting at the architectural level.
3. To enhance feature interaction, we design a patient-specific dynamic fusion strategy. Unlike traditional fixed-weight fusion, which cannot adapt to individual clinical variations, our approach enables fine-grained, personalized deep interaction between visual features and clinical metadata, thereby fully exploiting cross-modal associations.
4. To improve decision interpretability, we integrate Grad-CAM visualization to generate lesion activation heatmaps. This enables intuitive visualization of the model's attention regions, establishes traceable and verifiable diagnostic processes, and enhances clinical credibility.

Additionally, we construct a joint optimization and anti-overfitting framework that further enhances convergence stability and generalization performance. Our model achieves strong performance and ultra-high inference efficiency on public datasets, demonstrating strong potential for clinical translation.

## 2 Related work

### 2.1 Deep learning-based classification of skin lesions

Skin lesion classification represents a central research direction in intelligent medical image analysis. Its fundamental objective is to leverage deep learning techniques to automatically extract dermoscopic lesion features, thereby enabling accurate discrimination between benign and malignant lesions. Early research adopted convolutional neural networks (CNNs) as the foundational architecture. By stacking multi-scale convolutional layers and incorporating residual connections, these models effectively captured shallow visual features—including lesion texture, color, and boundary patterns—initially enabling automated diagnosis and classification of skin lesions. Esteva et al. (2) demonstrated that CNN-based skin cancer classification models achieve performance comparable to that of professional dermatologists, thereby validating the feasibility of CNNs for skin lesion diagnosis.

To address gradient vanishing and feature degradation in deep CNN training, He et al. (5) proposed Residual Network (ResNet), while Huang et al. (6) introduced the Densely Connected Network (DenseNet). These architectures primarily optimize feature propagation through skip connections, thereby improving the extraction efficiency and stability of deep networks and further enhancing skin disease diagnosis performance. However, CNNs suffer from an inherent inductive bias toward local receptive fields, which limits their ability to capture irregular boundary features. Furthermore, they exhibit insufficient discriminative capacity for small lesions, low-contrast lesions, and lesions in high-noise backgrounds, ultimately compromising the generalization of downstream models. The Swin Transformer, leveraging its hierarchical feature pyramid and shifted window multi-head self-attention mechanism, effectively overcomes the modeling limitations of CNNs. It efficiently captures global image context and multi-scale feature correlations while maintaining controlled computational complexity. It has demonstrated superior performance in the ISIC series of skin imaging challenges (8, 9). However, existing single-modal diagnostic models based on the Swin Transformer rely solely on visual information, entirely neglecting the auxiliary discriminative value of clinical metadata. Consequently, these models fail to generalize to complex clinical diagnosis and treatment scenarios. Their robustness and clinical practicality remain significantly limited. Therefore, integrating clinical metadata through multimodal fusion has emerged as the prevailing approach for enhancing Swin Transformer-based single-modal models.

### 2.2 Multimodal fusion in medical imaging

Medical multimodal fusion technology is designed for integrating multi-source heterogeneous information covering medical images, text, and structured clinical data. It explores the interrelationships across different modalities, compensates for feature deficiencies in single-modal data, and ultimately improves the diagnostic reliability and generalization capability of medical models. Currently, mainstream multimodal fusion methods can be categorized into three classes.

Early fusion directly fuses image features and structured metadata via feature concatenation, featuring simple structure, low computational complexity, and convenient deployment. However, image features typically have high dimensionality (ranging from hundreds to thousands of dimensions), while clinical metadata only contains dozens of features. Substantial discrepancies in scale, distribution and semantic space between the two types of data cause clinical prior knowledge to be submerged by high-dimensional visual features, failing to exert auxiliary effects. More critically, this approach may introduce heterogeneous noise that triggers negative transfer, ultimately leading to degraded model performance.

Late fusion, a decision-level fusion method, performs weighted fusion or voting-based fusion on the output results of independent models trained for each modality. It is characterized by low modal coupling and high stability during model training, and can effectively avoid the negative transfer problem that exists in early fusion. Nevertheless, late fusion lacks deep cross-modal feature interaction, which makes it unable to capture fine-grained correlations between image features and clinical information, and the complementarity of features from different modalities cannot be fully utilized. As a result, the improvement of model performance achieved by late fusion is limited.

Attention-driven deep fusion achieves fine-grained interaction of multimodal features through cross-attention and co-attention mechanisms, and its feature representation capability is significantly superior to the two aforementioned methods. However, this fusion method has a complex model structure with a large number of parameters. Combined with the inherent challenges of skin lesion clinical data, including sparse samples, high annotation costs and class imbalance, this method is extremely prone to severe overfitting, leading to outstanding performance on the training set but substantially degraded performance on the test set.

Based on existing research on attention-driven deep fusion, how to balance deep cross-modal feature interaction, model lightweighting, and overfitting suppression capability has become a key problem demanding urgent solutions in current multimodal intelligent diagnosis of skin lesions.

### 2.3 Hypernetworks and low-rank learning

Hypernetworks were initially proposed by Ha et al. (18) and formally published in 2017. The core concept of this method is to dynamically generate weight parameters for the main network via a lightweight auxiliary network (namely the hypernetwork) conditioned on the input data, thereby enabling personalized adaptive modeling for the target model. This architecture is a typical implementation paradigm of meta-learning, in which the hypernetwork generates parameters for the main network in real time in accordance with the input task, data or context information, allowing the main network to achieve flexible adaptation to diversified task scenarios. This mechanism strikes an excellent balance between parameter efficiency and model flexibility, rendering it particularly applicable to resource-constrained medical task scenarios. Nevertheless, conventional hypernetworks directly generate full-rank weight matrices, where the parameter scale and computational complexity grow quadratically with the increase of feature dimension, which leads to excessive computational overhead of the model and prolonged inference latency. Furthermore, excessive model capacity will trigger severe overfitting on small-sample medical datasets, restricting the clinical translation and application of related models.

Low-rank learning is a model lightweighting and feature optimization technique. Its most typical implementation, low-rank matrix decomposition, decomposes high-dimensional redundant weight matrices into the product of two low-rank matrices. On the premise of retaining critical feature information, this method achieves three simultaneous optimization effects: it substantially compresses the number of model weight parameters, reduces overall computational complexity, and suppresses overfitting by constraining model capacity, ultimately achieving significant acceleration of the model inference process (19). At present, low-rank matrix decomposition has been widely applied in fields including Transformer model lightweighting, large-scale model parameter compression, and medical image feature dimensionality reduction. With its efficient feature extraction and dimensionality compression capabilities, this technique provides robust support for algorithm optimization and performance improvement in various research fields, and has yielded outstanding results.

The dynamic combination of low-rank constraints and hypernetworks can simultaneously leverage the dual advantages of “parameter lightweighting + flexible adaptive adjustment”. On one hand, the hypernetwork generates small-scale weight parameters in accordance with low-rank constraints, which substantially reduces model complexity. On the other hand, low-rank parameters, serving as the “dynamic adjustment factor” of the model, enable the model to adaptively adjust feature representation based on the characteristics of input data. This allows the model to retain the high representation capability of complex models while ensuring lightweighting, and maximizes the precise balance between computational efficiency and representation capability. However, existing studies generally employ low-rank compression or hypernetwork-based dynamic weight generation in isolation, and lack coupled architectural designs that integrate the two methods for multimodal fusion. As a result, existing methods cannot achieve dynamic deep interaction between cross-modal features while ensuring model lightweighting, and ultimately fail to simultaneously satisfy the core clinical requirements of high diagnostic accuracy, model lightweighting, and high robustness. On this basis, this study integrates the respective advantages of low-rank learning and hypernetworks to construct a lightweight dynamic multimodal fusion architecture, which provides a novel paradigm for multimodal learning with small-sample medical images.

## 3 Methods

This section establishes a complete technical framework for the multimodal skin lesion classification task, covering data preprocessing, model architecture design, core fusion mechanism, training strategy and interpretability verification. Aiming at the prevalent limitations of existing methods, including cross-modal negative transfer, overfitting under small-sample conditions, insufficient cross-modal feature interaction and opaque decision-making processes, this study proposes the Gated Low-Rank Hyper-Fusion Network (GLHF-Net). The proposed method adopts lightweight Swin Transformer (Swin-T) as the visual backbone, and follows an architecture of “purification before fusion”. Specifically, a metadata-driven gating mechanism is designed to suppress cross-modal interference, and a low-rank hyper-fusion module is employed to achieve lightweight deep cross-modal feature interaction. A joint optimization strategy is further integrated to improve the generalization performance of the model. Finally, Grad-CAM is introduced to realize decision visualization, which provides a complete technical solution for intelligent skin lesion diagnosis that balances classification accuracy, inference efficiency and decision reliability. The overall experimental and evaluation process is presented in Figure 1.

**Figure 1:**
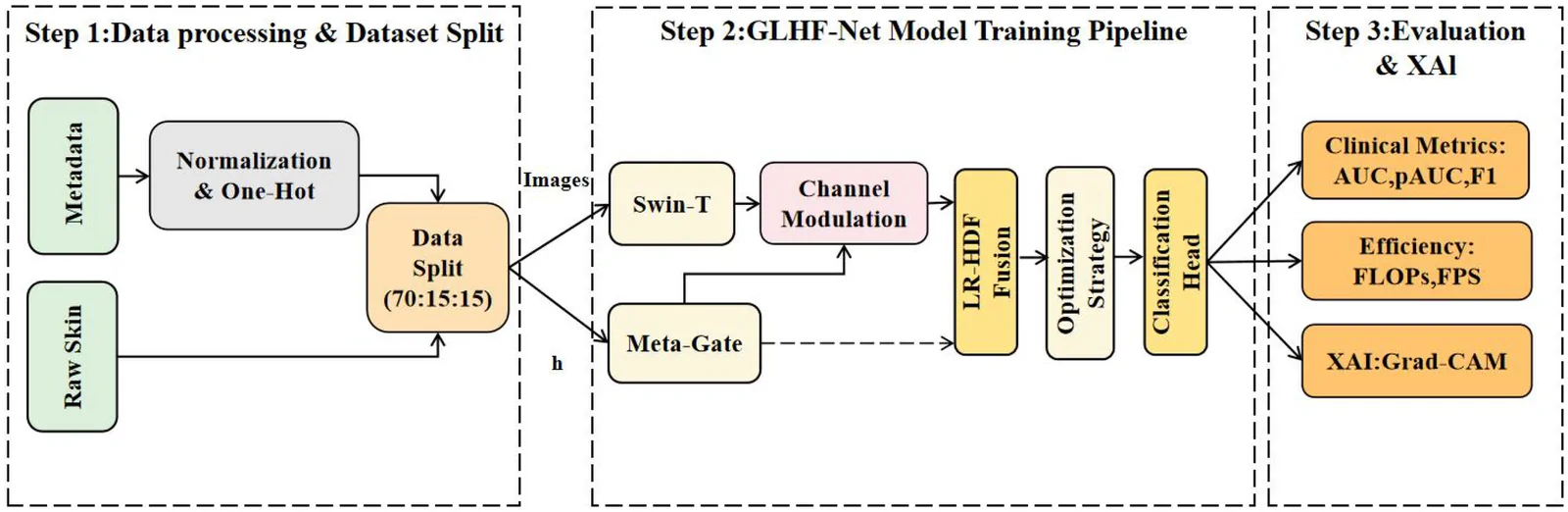
Overall experimental and evaluation process.

The experimental workflow of the proposed GLHF-Net includes three consecutive steps:

Step 1: Data processing and dataset partitioning. Original skin lesion images are processed via ROI mask extraction and data augmentation to obtain standardized lesion images with size 224 × 224 × 3; original clinical metadata are converted into a 64-dimensional latent clinical vector h through min-max normalization and one-hot encoding. To avoid cross-patient data leakage, all samples are partitioned into training, validation, and test sets at the ratio of 70:15:15 based on patient IDs;
Step 2: GLHF-Net training. Preprocessed lesion images are input into the Swin-T backbone network to extract 768-dimensional global visual features (Fimg); the Meta-Gate module takes the latent clinical vector (h) as input and outputs two branches: one branch generates channel attention masks (α) for channel modulation to obtain optimized visual features (Fgated), while the other branch inputs h into the LR-HDF fusion module for dynamic weight generation. After the purified visual features Fgated and the clinical vector h are fused via the LR-HDF module, the fused features are processed by a two-layer linear classification head with dropout normalization. Finally, the model is optimized with the focal loss function and the differential learning rate strategy;
Step 3: Quantitative evaluation and XAI interpretability analysis. Multi-dimensional evaluation is conducted, covering clinical diagnostic metrics (AUC, pAUC, *F*1), model inference efficiency (FLOPs, FPS), and interpretability analysis based on Grad-CAM visualization.

### 3.1 Data and preprocessing

#### 3.1.1 Dataset construction and clinical re-annotation

This study utilizes the HAM10000 dataset (22), a standard public dataset widely adopted in international dermatological imaging research. The dataset comprises 10,015 dermoscopic images, each of which is paired with structured clinical metadata including patient age, gender, and anatomical location of the lesion, and covers seven common clinical types of skin lesions. To align with the practical clinical requirements of early skin lesion screening and biopsy risk stratification, this study transforms the original multi-classification task into a clinically more valuable benign-malignant binary classification task, and conducts category division strictly following the clinical risk stratification criteria in dermatology:

Malignant group: Melanoma (MEL), Basal Cell Carcinoma (BCC), Actinic Keratosis (AK);

Benign group: Melanocytic Nevus (NV), Benign Keratosis Lesion (BKL), Dermatofibroma (DF), Vascular Lesion (VASC).

Actinic Keratosis (AK) was grouped into the malignant category because it is a squamous intraepithelial neoplasia, i.e., an *in situ* form of squamous cell carcinoma, that carries well-documented potential to progress to invasive squamous cell carcinoma; classifying AK as malignant therefore better aligns with the clinical goal of early screening and biopsy risk stratification.

This classification framework preserves the intrinsic properties of real-world clinical data inherent to the dataset, including class imbalance, uneven sample distribution, high morphological heterogeneity of lesions, and complex noise interference. Consequently, the model training and evaluation scenarios constructed in this study are highly consistent with real clinical screening settings, which effectively guarantees the clinical generalizability of the proposed model.

#### 3.1.2 Dermoscopic image preprocessing and ROI mask denoising

To mitigate noise interference including hair, bubbles, illumination artifacts and redundant background in dermoscopic images, and to prevent the model from learning invalid pseudo-features and producing feature bias in the training process, this study incorporates a Hard ROI Masking strategy at the image preprocessing stage. First, a single-channel binary lesion mask that is precisely aligned with the original image is acquired. Next, the dimension of the mask is expanded into a three-channel matrix to match the dimension of the original RGB image, and element-wise bitwise AND operation is performed between the original image and the mask matrix. This operation is mathematically equivalent to the Hadamard product (element-wise multiplication) operation. The calculation formula is presented as follows:

$$I_{\mathrm{ROI}}=I⊙M$$ (1)

In Equation 1: I denotes the original dermatoscopic input image scaled to a resolution of 224 × 224, and $M\in \{0,1{\}}^{H\times W\;}$ is the binarized lesion mask matrix. The resulting $\;I_{\mathrm{ROI}}$ has its background pixels forcibly set to zero, achieving a pure black masking effect, while preserving all texture details and key pathological features of the lesion region intact. This preprocessing step effectively blocks the propagation path of background noise at the data input stage, enabling the subsequent visual backbone network to focus on extracting fine representations specific to the lesions.

#### 3.1.3 Clinical metadata encoding and normalization

Clinical metadata comprises continuous and categorical features, and suffers from issues including inconsistent feature dimensions, substantial variations in value distribution, and unordered categorical attributes. Directly inputting unprocessed metadata into the model will induce training oscillation and result in unbalanced feature weights. Accordingly, this study adopts differentiated standardization procedures for distinct types of metadata to guarantee that the input data is unbiased, dimensionally aligned and effective. For the continuous variable age, the Min-Max normalization method is employed to uniformly map all values to the interval [0,1] for eliminating dimensional discrepancies. The calculation formula is presented as follows:

$$x_{\mathrm{age}}^{\mathrm{norm}}=\frac{x_{\mathrm{age}}-\min(X_{\mathrm{age}})}{\max(X_{\mathrm{age}})-\min(X_{\mathrm{age}})}$$ (2)

In Equation 2: $x_{\mathrm{age}}\;$ is the original age value $\;X_{\mathrm{age}}$ is the age dataset of all samples, $x_{\mathrm{age}}^{\mathrm{norm}}$ is the normalized age feature.

For unordered categorical variables including gender and anatomical site, One-Hot Encoding is adopted to transform such variables into orthogonal binary sparse vectors. This approach completely eliminates the pseudo-ordinal relationship introduced by label encoding, thereby guaranteeing the objectivity of metadata representation. Ultimately, all preprocessed clinical features are concatenated into a fixed-dimensional dense feature vector, $x_{\mathrm{meta}}\in R^{K}$ which provides stable and standardized clinical prior input for the subsequent gating mechanism and hyper-fusion module.

In clinical practice, structured metadata such as anatomical site and age are frequently missing or recorded as unknown. To ensure that the model remains applicable when metadata is incomplete, the preprocessing pipeline explicitly handles missing values as follows. For the continuous variable age, missing values are imputed with the median value of the training set before Min–Max normalization. For the categorical variables sex and anatomical site, missing or unknown values are encoded as a dedicated “unknown” category within the one-hot encoding. In this way, the model explicitly learns a representation for missing information instead of discarding such samples or introducing systematic bias, which is consistent with real-world clinical screening scenarios.

After finishing data preprocessing and initialization, this study conducts data partitioning strictly based on patient ID, which fundamentally eliminates the interference of homologous image data leakage on model performance evaluation. The specific partitioning scheme is as follows: for the HAM10000 cohort including 7,470 patients, this study strictly controls the proportion of patients in the training set, validation set, and independent test set to 70% (5,229 patients), 15% (1,120 patients), and 15% (1,121 patients), respectively.

### 3.2 Overall architecture of gated low-rank hyper-fusion network (GLHF-Net)

Aiming at the multimodal auxiliary diagnosis task of skin lesions, this study proposes a lightweight Gated Low-Rank Hyper-Fusion Network (GLHF-Net). The model adopts a two-stage progressive fusion architecture, and strictly follows the design paradigm of “denoising and purification first, dynamic fusion later”. In this model, a lightweight Swin-T is employed as the visual backbone network to extract high-dimensional visual features $F_{\mathrm{img}}\in R^{768}$. To fully utilize clinical prior knowledge, the standardized clinical metadata $x_{\mathrm{meta}}$ is encoded and input into the multimodal fusion module together with the extracted visual features. The overall forward propagation process of the model can be uniformly represented by the following composite function:

$$P_{\mathrm{malignant}}=H_{\mathrm{cls}}(F_{\mathrm{LR}\text{-}\mathrm{HDF}}(F_{\mathrm{Gate}}(F_{\mathrm{img}},x_{\mathrm{meta}}),x_{\mathrm{meta}}))$$ (3)

In Equation 3: $F_{\mathrm{Gate}}$ corresponds to the metadata feature purification module, which is responsible for suppressing inter-modal negative transfer and purifying visual features; $F_{\mathrm{LR}\text{-}\mathrm{HDF}}$ denotes the LR-HDF low-rank dynamic fusion module, which is responsible for realizing deep interaction between different modal features and completing lightweight modeling; $H_{\mathrm{cls}}$ denotes the classification prediction head, and $P_{\mathrm{malignant}}$ represents the predicted probability that the output lesion is malignant.

The overall architecture of the proposed model and the evolution details of tensor dimensions are presented in Figures 2, 3 respectively, which explicitly illustrate the parameter configuration and dimension transformation rules across the full processing pipeline from Patch Embedding, Swin Block, metadata encoding, gating-based purification, low-rank fusion to classification output.

**Figure 2:**
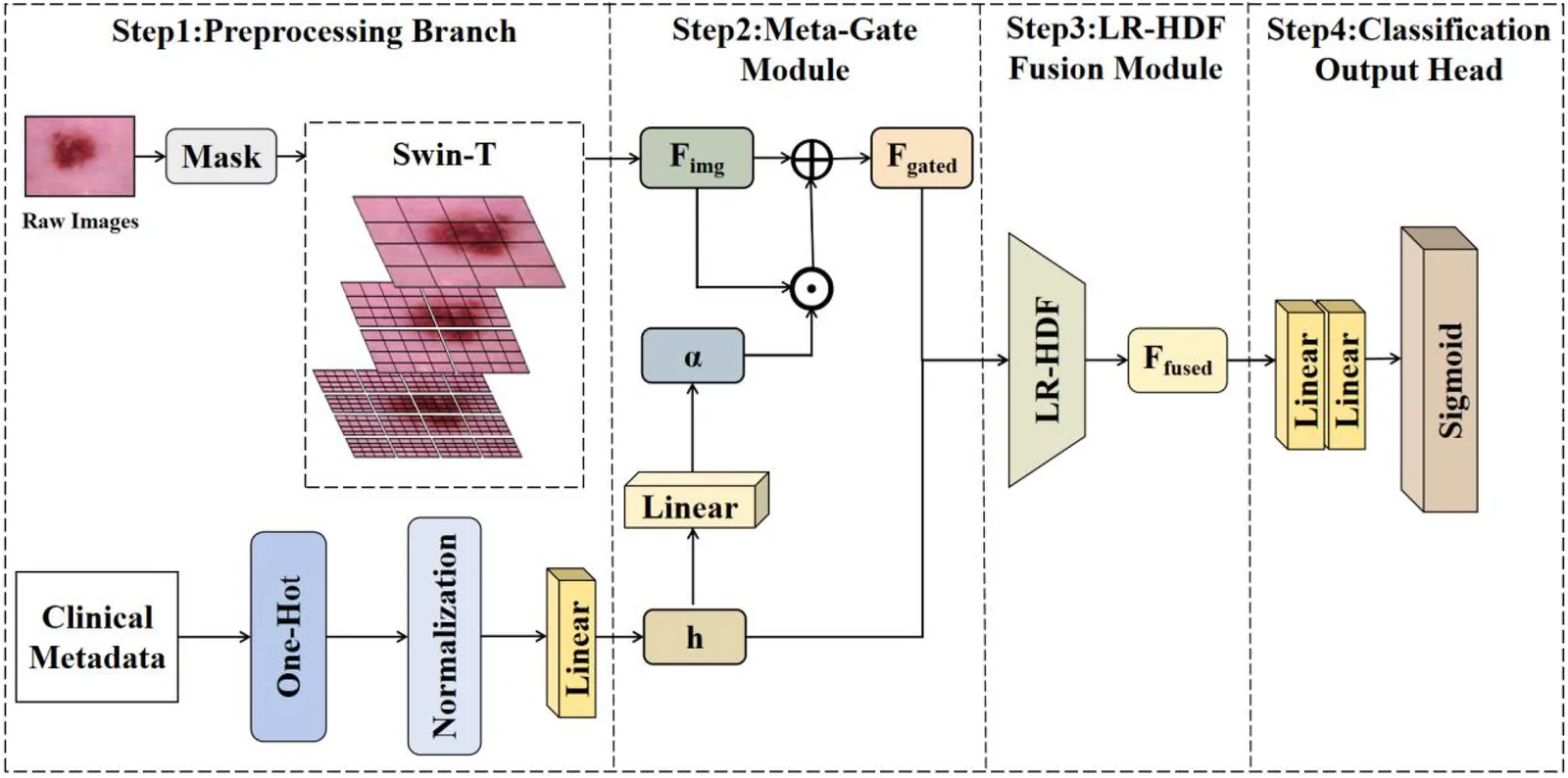
Overall architecture of GLHF-Net.

**Figure 3:**
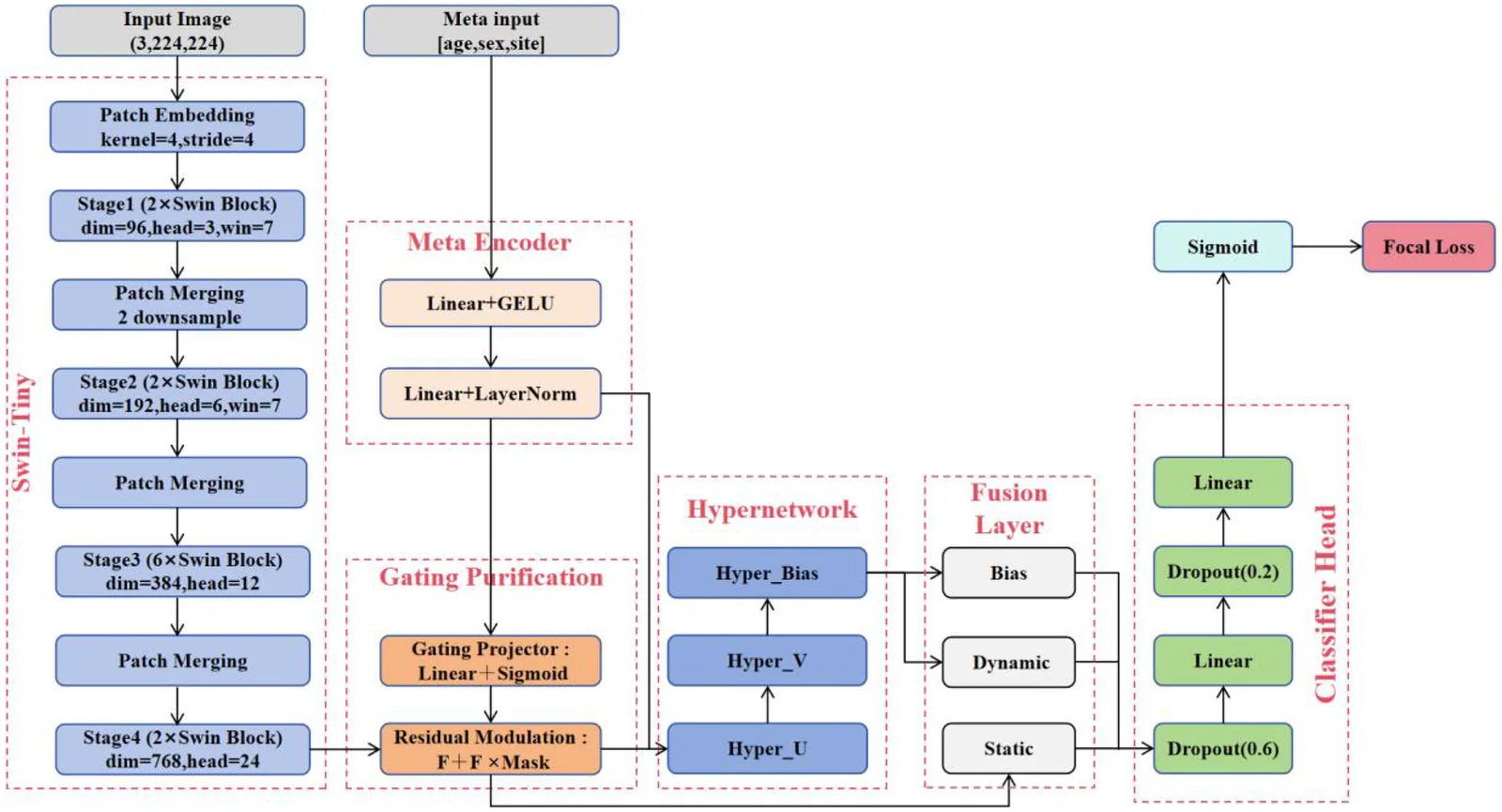
GLHF-Net network engineering details and tensor dimension evolution diagram.

Figure 2 illustrates the overall architecture of GLHF-Net, which consists of four sequential processing stages. Stage 1 corresponds to the bimodal preprocessing branch: the original 224 × 224 × 3 dermatoscopic image is subjected to background artifact removal via Hadamard product operation with the ROI lesion mask, and then input into Swin-T to extract a 768-dimensional global visual feature vector Fimg; structured clinical metadata are processed sequentially via one-hot encoding, normalization and Meta Encoder encoding to generate a fixed 64-dimensional clinical latent vector h. Stage 2 implements the Meta-Gate metadata gating module: the clinical vector h is processed through a linear layer to generate a 768-dimensional channel attention mask α, and channel-level purification of visual features is realized via the residual weighting formula $F_{\mathrm{gated}}=F_{\mathrm{img}}⊕(F_{\mathrm{img}}⊙\alpha )$, thereby alleviating the problem of multimodal negative transfer. Stage 3 is the LR-HDF low-rank hyper-dimensional fusion module, which takes the purified gated visual feature Fgated and the clinical latent vector h as co-inputs, and outputs a 256-dimensional multimodal fusion feature Ffused. Stage 4 is the classification output stage: the fusion feature is processed through two linear network layers with Dropout regularization (*p* = 0.6), and finally activated via the sigmoid function to obtain the prediction probability of malignant skin lesion progression.

Figure 3 illustrates the evolution of tensor dimensions of the GLHF-Net model throughout the complete processing pipeline, ranging from input images and clinical metadata to the final classification output. For the six core modules, namely Patch Embedding, Swin Block, Meta Encoder, Gating Purification, Hypernetwork Fusion, and Classifier, the parameter configurations, tensor dimension transformation rules and core operation logic are all marked explicitly. This diagram presents the full-path tensor flow details from feature extraction, gating modulation, low-rank fusion to classification output under the lightweight design framework of the proposed model.

### 3.3 Gating purification and low-rank hyper-fusion mechanism

The core innovations of this study consist of the Meta-Gate channel-wise gating purification mechanism and the LR-HDF low-rank hyper-fusion module. The two modules operate in a collaborative manner and address the deficiencies of existing methods in a hierarchical manner: the gating mechanism undertakes the task of pre-denoising and prevents cross-modal negative transfer, while the low-rank hyper-fusion module is responsible for enabling deep cross-modal feature interaction and realizing lightweight anti-overfitting. Together, the two modules jointly construct a multimodal fusion architecture with high precision, high robustness and lightweight characteristics.

#### 3.3.1 Metadata-driven channel-wise gating mechanism

In traditional multimodal early fusion frameworks, high-dimensional image features are directly concatenated with low-dimensional metadata. This practice is highly susceptible to issues including metadata signal dilution, noise superposition, and semantic inconsistency, ultimately inducing the problem of cross-modal negative transfer. To address this limitation, this study proposes the Meta-Gate channel-wise gating mechanism, which leverages clinical metadata to guide the screening of visual features prior to multimodal fusion. This mechanism achieves lesion feature enhancement, noise suppression, and semantic alignment, thereby effectively mitigating negative transfer across modalities.

##### 3.3.1.1 Metadata encoding and latent vector generation

The standardized clinical metadata vector $x_{\mathrm{meta}}$ is characterized by sparse dimensions and shallow semantic expression, and cannot directly guide the modulation of high-dimensional visual features. Accordingly, this study constructs a lightweight encoder, through which the input metadata vector $x_{\mathrm{meta}}$ is processed via linear transformation, normalization and activation function mapping to generate a low-dimensional dense latent vector (i.e., the compressed representation of clinical information) with unified dimensions that retains the original clinical information, thereby realizing the deep representation of the original clinical prior knowledge. The corresponding calculation formula is presented as follows:

$$h=\mathrm{LayerNorm}(\mathrm{GELU}(W_{1}x_{\mathrm{meta}}+b_{1}))$$ (4)

In Equation 4: $W_{1}\in R^{K\times 64}$, b1∈R64 are the learnable weight matrix and bias vector corresponding to clinical metadata, respectively; $h\in R^{64}$ denotes the low-dimensional latent vector encoding the prior clinical information of the patient. This study adopts the combined architecture of LayerNorm normalization and GELU activation function, and its core functions are reflected in two aspects: on one hand, this combination can stabilize the overall feature distribution of the latent vector, constrain the numerical range of features within a reasonable interval during network training, and thus guarantee the numerical stability of the training process; on the other hand, the smooth nonlinear property of the GELU activation function can render the feature output of latent vectors smoother, effectively alleviate the gradient oscillation problem encountered in model training, and ultimately improve the convergence efficiency and training stability of the model. Specifically, the K-dimensional standardized clinical metadata vector x_meta_ is mapped to the 64-dimensional low-dimensional latent vector h through the learnable affine transformation parameterized by the weight matrix W_1_ and bias vector b_1_, whose dimensions are K × 64 and 64, respectively.

##### 3.3.1.2 Generation of channel-wise attention masks

To achieve precise channel-wise modulation of high-dimensional visual features using low-dimensional clinical latent vectors, this study conducts dimensional mapping on the patient clinical latent vector h to generate a channel-wise soft attention mask α that is fully dimensionally aligned with the visual feature channels of the Swin-T model. This mask constitutes a model-based adaptive weight matrix, which can quantify the contribution of each channel's visual features to lesion diagnosis based on the patient's personalized clinical information. Accordingly, this approach assigns differentiated weights to visual features of each channel, enabling accurate screening of effective pathological features and suppression of invalid noise features. The calculation formula is presented as follows:

$$\alpha =\sigma (W_{2}h+b_{2})\in {\left(0,1\right)}^{d}$$ (5)

In Equation 5: $W_{2}\in R^{64\times d}\mathrm{and}\;b_{2}\in R^{d}\;$ are learnable parameters; d denotes the number of visual feature channels; α is the soft attention mask; σ(⋅) corresponds to the Sigmoid activation function, which can constrain the values of the output attention mask α to the open interval (0,1). A mask value closer to 1 indicates that the corresponding channel contributes more to lesion diagnosis, while a mask value closer to 0 indicates that the channel is more likely to be identified as a redundant noise channel.

##### 3.3.1.3 Channel weighting and residual fusion

Based on the generated soft attention mask α, channel-wise recalibration is implemented on the importance of the original visual features. The core objective of this operation is to enhance the weights of visual channels carrying effective information and suppress invalid channels that only contain redundant noise. The introduction of residual connections facilitates the preservation of the original global visual features, which not only guarantees the integrity of the original feature information, but also effectively alleviates the gradient vanishing problem in the model training process. The calculation formula for channel-wise feature importance calibration is given as follows:

$$F_{gated}=F_{img}⊕(F_{img}⊙\alpha )$$ (6)

In Equation 6: ⊙ denotes the channel-wise element-wise multiplication operation, α represents the soft attention mask, through which channel-wise calibration of visual features is performed via $F_{\mathrm{img}}⊙\alpha$; ⊕ denotes the residual element-wise addition operation. $F_{\mathrm{gated}}$ is the gated-purified visual feature, which only retains lesion information strongly correlated with clinical diagnosis and effectively eliminates the interference from invalid channel information.

#### 3.3.2 Low-rank hyper-dynamic fusion (LR-HDF) module

Traditional full-rank hypernetworks directly generate complete high-dimensional weight matrices, where the number of parameters scales quadratically with feature dimension. This characteristic significantly increases the risk of severe overfitting in small-sample learning scenarios; furthermore, fixed global weights cannot accommodate the inter-individual clinical heterogeneity among patients. In this work, we integrate low-rank matrix decomposition with the dynamic weight generation mechanism of hypernetworks to construct the proposed LR-HDF module. Specifically, low-rank constraints are introduced to compress the parameter scale and reduce model complexity, and the hypernetwork is driven to generate patient-specific dynamic weights based on the clinical latent vector h, which achieves lightweight model deployment and personalized feature fusion. This design effectively suppresses overfitting while significantly improving the clinical adaptability and prediction performance of the model. The overall workflow of the LR-HDF module is illustrated in Figure 4.

**Figure 4:**
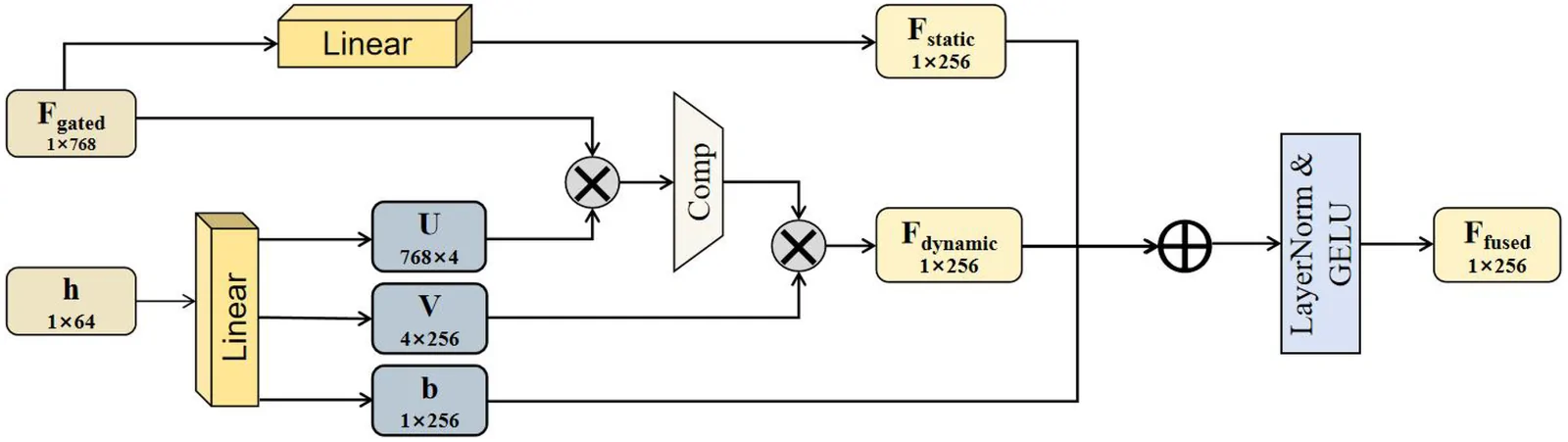
Low-rank hyper-dynamic fusion flowchart.

Figure 4 illustrates the tensor dimension transformation process and the core operational logic of the proposed LR-HDF module. The module takes the gated visual feature $F_{\mathrm{gated}}\in R^{1\times 768}$ and the clinical latent vector $h\in R^{1\times 64}$ as inputs, and is structured into two parallel feature extraction branches:

The static feature branch: This branch extracts general, dataset-level lesion features by passing F_gated_ through a standard linear projection layer, outputting the static feature representation F_static_∈R^1^ ^×^ ^256^.

The dynamic low-rank fusion branch: Conditioned on the clinical metadata h, a lightweight hypernetwork first generates the low-rank decomposition matrices U ∈ R^768^ ^×^ ^4^, V ∈ R^4^ ^×^ ^256^, and the dynamic bias term b ∈ R^1^ ^×^ ^256^. Two consecutive matrix multiplication operations are then performed on F_gated_ to complete the low-rank compression (yielding an intermediate representation) and reconstruction, thereby obtaining the personalized dynamic feature Fdynamic ∈ R^1^ ^×^ ^256^.

Finally, the static feature $F_{\mathrm{static}}$, the dynamic feature $F_{\mathrm{dynamic}}$, and the dynamic bias b are aggregated via element-wise addition. After feature optimization through LayerNorm and GELU activation, the module outputs the final 256-dimensional multimodal fused feature $F_{\mathrm{fused}}$.

##### 3.3.2.1 Low-rank weight decomposition and patient-specific weights

Traditional fully-connected fusion layers and full-rank hypernetworks are required to generate high-dimensional weight matrices, which suffer from large parameter scales, substantial redundant features and significantly high overfitting risks. The proposed LR-HDF module introduces a low-rank constraint [Rank r ≪ min(d_in,_ d_out_)], which decomposes the dynamic weight matrix into the product of two low-rank matrices Wdyn = UV, thus achieving substantial compression of the parameter scale. Meanwhile, based on the clinical latent vector h, this study generates exclusive personalized weights and biases for each patient via a lightweight hypernetwork to realize adaptive fusion. The corresponding calculation formulas are presented as follows:

$$U=\Phi_{U}(h)\in R^{d_{\mathrm{in}}\times r}$$ (7)

$$V=\Phi_{V}(h)\in R^{r\times d_{\mathrm{out}}}$$ (8)

$$b=\Phi_{b}(h)\in R^{d_{\mathrm{out}}}$$ (9)

In Equations 7–9: $\Phi_{U}$, $\Phi_{V}$, and $\Phi_{b}$ denote lightweight hypernetworks with a single-layer linear mapping structure (no intermediate hidden layers). They generate the dynamic low-rank matrices U, V and the adaptive bias term b, respectively. This lightweight design ensures efficient weight generation without introducing extra computational overhead.

##### 3.3.2.2 Dynamic low-rank feature reconstruction

Based on the low-rank matrix generated by the hypernetwork, the LR-HDF method executes two-step sequential Batch Matrix Multiplication (BMM) operations on the preprocessed visual features $F_{\mathrm{gated}}$. The aforementioned reconstruction process can be formulated as follows:

$$F_{dynamic}=(F_{gated}U)V$$ (10)

Equation 10 corresponds to the latter two core operations: low-rank compression and feature reconstruction. The first step is low-rank feature compression: under the constraint of the low-rank hyperparameter r, the preprocessed high-dimensional visual features with a dimension of 1 × 768 are compressed into core features with a dimension of 1×r. This operation enables the model to eliminate redundant noise and invalid features, retain task-relevant core feature information, and achieve overfitting suppression for small-sample scenarios at the model structure level. The second step is personalized feature reconstruction: the obtained low-dimensional core features are reconstructed into dynamic fusion features with a dimension of 1 × 256. While restoring the feature representation dimension, this module integrates patient-specific clinical heterogeneity, and realizes fine-grained deep interaction among multimodal features.

##### 3.3.2.3 Parameter complexity and fusion features

In the architectural design of this study, when the traditional full-rank weight matrix is adopted, the number of parameters of the fusion layer is 768×256=196,608; After introducing the low-rank constraint (r=4), the parameter complexity reduces to $r\times (d_{\mathrm{in}}+d_{\mathrm{out}})=4\times (768+256)=4,096$. Through low-rank decomposition, the number of parameters of the model at the fusion layer decreases sharply by 97.9%, which not only significantly reduces GPU memory and computational overhead, but also establishes a rigorous implicit regularization constraint.

To simultaneously incorporate the general feature distribution of the entire dataset and the individual-specific features of each patient, this study aggregates the static global projection branch and the dynamic personalized reconstruction branch in a joint manner to output the final high-quality full-volume multimodal fusion feature $F_{\mathrm{fused}}$. The corresponding calculation formula is presented as follows:

$$F_{\;fused}=GELU(LayerNorm(F_{gated}W_{static}+F_{dynamic}+b))$$ (11)

In Equation 11: $W_{\mathrm{static}}$ serves as the static base network, which undertakes the task of extracting common lesion morphological features shared across all samples; $F_{\mathrm{dynamic}}$ denotes patient-specific dynamic features, which are responsible for capturing heterogeneous pathological features of individual patients; the dynamic bias term b is applied to correct distribution drift among different samples. Finally, feature optimization is achieved via LayerNorm normalization and GELU nonlinear activation, and the high-quality fused feature $F_{\mathrm{fused}}$ is output.

### 3.4 Training objectives and joint optimization strategies

#### 3.4.1 Loss function: focal loss

The category distribution of the HAM10000 dataset exhibits a significant long-tailed characteristic, which is specifically reflected in the fact that the number of malignant samples is substantially smaller than that of benign samples. The conventional cross-entropy loss tends to bias model prediction toward benign samples, which ultimately leads to missed diagnosis of malignant samples. To solve this problem, this study introduces the Focal Loss function, which can adaptively adjust the loss weight according to the classification difficulty of each sample. Specifically, this method reduces the contribution of easily classified benign samples to the total loss, while increasing the weight proportion of difficult-to-classify malignant samples, which directs the model training to focus on feature learning for difficult samples, and consequently improves the predictive performance of the model for malignant samples. The formula of the loss function is given as follows:

$$L_{\;focal}=-\sum_{i=1}^{N}\left[y_{i}\alpha_{t}{\left(1-p_{i}\right)}^{\gamma }\log\;(p_{i})+(1-y_{i})(1-\alpha_{t})p_{i}^{\gamma }\log\;(1-p_{i})\right]$$ (12)

In Equation 12: N denotes the total number of samples, $y_{i}$ represents the ground-truth label of the i-th sample, $p_{i}$ is the malignant probability predicted by the model for the i-th sample. In this study, the adjustment factor γ is set to 2.0, which can exponentially reduce the contribution of easily-classified samples to the overall gradient, thereby forcing the optimization direction of the network to shift toward difficult-to-classify lesions with high information entropy. Meanwhile, this study introduces the balance factor $\alpha_{t}$ set to 0.25 to alleviate the inherent class imbalance between positive and negative samples. The synergistic effect of the two hyperparameters substantially reduces the missed diagnosis risk of malignant lesions starting from the design of the optimization objective.

#### 3.4.2 Adoption of differential learning rate strategy

To prevent the unified learning rate from disrupting the underlying feature distribution learned by the backbone network and accelerate the parameter convergence of the newly added fusion modules, this study adopts a hierarchical differential learning rate strategy. Specifically, the learning rate of the Swin-T backbone network is set to $1\times 10^{-5}$ to avoid that a large learning rate damages the general visual features already learned by the backbone, thereby preserving its well-learned feature extraction capability; meanwhile, the learning rate of newly added modules including Meta-Gate, LR-HDF, and classification heads is set to $2\times 10^{-4}$. This differential updating mechanism accelerates the parameter convergence of newly added modules via a larger update step size, and realizes a trade-off between the preservation of pre-trained knowledge and the rapid optimization of newly added modules, which guarantees the hierarchical and accurate training of the model.

#### 3.4.3 Joint optimization and anti-overfitting system

To further enhance the anti-overfitting capability of the model and adapt to small-sample medical data training scenarios, this study constructs a multi-dimensional joint optimization and anti-overfitting framework: a high-rate Dropout mechanism with *p* = 0.6 is employed to randomly inactivate neurons in the classification head, thereby suppressing overfitting of high-level features; a weight decay coefficient of 0.05 is set to constrain the magnitude of model parameters and prevent excessive parameter updating; a cosine annealing learning rate scheduling strategy is introduced to realize fine-grained convergence in the late training phase and improve the accuracy of the obtained optimal solution; the early stopping patience coefficient is configured as 15, which monitors the model performance on the validation set and terminates invalid training in a timely manner to effectively inhibit overfitting. The combination of multiple regularization strategies ensures stable model training, efficient convergence, and excellent generalization performance.

### 3.5 Interpretability mechanism

Breaking the “black-box decision-making” constraint of deep neural networks in computer-aided medical diagnosis is a core prerequisite for satisfying the safety supervision requirements of medical artificial intelligence and improving clinicians' acceptance of intelligent auxiliary diagnosis systems. Accordingly, this study proposes Gradient-weighted Class Activation Mapping (Grad-CAM) at the decision output layer to establish a visual validation module for the model's decision logic. This approach acquires the spatial gradient of the target category with respect to the deep feature map via backpropagation, and further generates a class activation heatmap that can intuitively characterize the attention distribution of the model. The corresponding mathematical derivation formula is presented as follows:

$$\alpha_{k}^{c}=\frac{1}{Z}\sum_{i}\sum_{j}\frac{\partial y^{c}}{\partial A_{ij}^{k}}$$ (13)

$$L_{Grad-CAM}=ReLU\left(\sum_{k}\alpha_{k}^{c}A^{k}\right)$$ (14)

In Equations 13, 14: $\alpha_{k}^{c}$ denotes the Grad-CAM importance weight of the k-th channel for target class c, obtained by global average pooling (with the normalization constant Z equal to the spatial size of the feature map) of the gradient of the model's predicted score yᶜ for class c with respect to the activation value $A_{\mathrm{ij}}^{k}$ at spatial position (i, j) of the k-th feature channel, where i and j are the row and column indices and c is the class index. This weight accurately quantifies the contribution of each channel to the final diagnostic result. In Equation 14, the class activation heatmap L_Grad−CAM_ is generated by linearly combining all channel maps Aᵏ weighted by $\alpha_{k}^{c}$ and applying ReLU to suppress gradients that negatively affect the target class, ensuring the heatmap highlights only regions that positively promote benign-malignant classification.

By overlaying the generated $L_{\mathrm{Grad}-\mathrm{CAM}}$ heatmap onto the original lesion image, the spatial attention distribution of the GLHF-Net model during diagnostic inference can be visually presented. If the high-response activation region of the model is highly consistent with the core pathological features that dermatologists focus on in clinical diagnosis (e.g., the classic ABCD rule: asymmetry, irregular border, uneven color, and specific lesion structure), it can be verified from both morphological and pathological perspectives that the multimodal fusion model proposed in this study does not blindly fit background noise or dataset artifacts, but effectively extracts and captures the deep pathological association rules behind the data. This interpretability mechanism not only provides solid evidence-based support for the model's prediction results, but also significantly improves the feasibility of translating the GLHF-Net architecture into real clinical application scenarios.

We acknowledge the inherent limitations of Grad-CAM saliency maps. Specifically, Grad-CAM produces class-activation heatmaps at a relatively coarse spatial resolution, can be sensitive to gradient noise, and may exhibit class-discrimination ambiguity for visually similar lesions; moreover, saliency maps reflect the model's attention rather than causal evidence of disease. Accordingly, the heatmaps presented in Figure 7 are intended as qualitative aids that support—but do not replace—clinician judgment, and we recommend that clinical decisions integrate the model output with the original dermoscopic images and patient history. A quantitative evaluation of the heatmaps (e.g., localization metrics or perturbation-based insertion/deletion tests) is an important direction of our future work.

## 4 Experiments

### 4.1 Implementation details and evaluation metrics

This study is implemented based on the PyTorch 2.1.0 deep learning framework under the Python 3.12 runtime environment. The hardware computing platform is equipped with a single NVIDIA RTX 5070 GPU with 12 GB of GDDR6 video memory. The AdamW optimizer is adopted, and a hierarchical differential learning rate strategy is applied to balance the preservation of pre-trained weights and the convergence speed of newly added modules: the learning rate of the Swin-T visual backbone is set to $1\;\mathrm{times}10^{-5}$, while the learning rate of newly added fusion modules including Meta-Gate, LR-HDF and classification heads is set to $2\;\mathrm{times}10^{-4}$. A cosine annealing strategy is employed for learning rate scheduling, with weight decay set to 0.05; the training batch size is set to 8. All experimental groups uniformly adopt Automatic Mixed Precision (AMP) training and an early stopping strategy with patience set to 15: the training process is terminated if the validation set AUC does not improve for 15 consecutive epochs, and the maximum number of training epochs is set to 30. To ensure the fairness and comparability of ablation experiments, all models are configured with identical optimizer settings, learning rate scheduling schemes and training protocols, with only the network structure modified.

This study adopts multi-dimensional quantitative indicators to evaluate model performance, which can be categorized into three groups: classification effectiveness indicators (AUC, pAUC, Acc, Precision, Recall, *F*1), hardware efficiency indicators (parameter count Params, computational complexity FLOPs, inference frame rate FPS), and clinical diagnostic indicators (Sens, Spec, PPV, NPV). Among these, pAUC, defined as the area under the ROC curve for TPR ≥ 0.8, is a core strict indicator for clinical diagnosis, which directly reflects the discriminative ability of the model under high sensitivity requirements. Additionally, four core clinical indicators derived from the confusion matrix are introduced, where TP, TN, FP, and FN denote true positive, true negative, false positive, and false negative, respectively, with their mathematical definitions provided in Equations (15–18):

Sensitivity:

$$Sens=\frac{TP}{TP+FN}$$ (15)

Specificity:

$$Spec=\frac{TN}{TN+FP}$$ (16)

PPV:

$$PPV=\frac{TP}{TP+FP}$$ (17)

NPV:

$$NPV=\frac{TN}{TN+FN}$$ (18)

### 4.2 Core ablation experiments

To identify the optimal visual backbone network tailored for the skin lesion classification task, this study compares the classification performance of mainstream convolutional neural network architectures (ResNet18, ResNet50, EfficientNet) and Transformer-based architectures (ConvNeXt_tiny, Swin-T) on single-modal dermoscopic image datasets. The corresponding experimental results are presented in Table 1.

**Table 1:** Performance comparison of each model.

Model configuration	pAUC	AUC	Sensitivity	Specificity	*F*1-score
ResNet18	0.6447	0.8757	0.8006	0.7682	0.5817
ResNet50	0.6501	0.8893	0.7422	0.8552	0.6352
EfficientNet	0.6644	0.8848	0.6804	0.8744	0.6197
ConvNeXt-tiny	0.6802	0.8955	0.7422	0.8410	0.6197
Swin-T	0.6811	0.9071	0.7457	0.8778	0.6636

Experimental results demonstrate that Swin-T outperforms ResNet series, EfficientNet and ConvNeXt-tiny in terms of global AUC, pAUC and *F*1 score, and attains the optimal trade-off between sensitivity and specificity. Although ResNet18 achieves the highest sensitivity, it exhibits low specificity, which tends to induce a large number of false positive predictions. By contrast, EfficientNet delivers satisfactory specificity but insufficient sensitivity, failing to satisfy the core requirement of “avoiding missed diagnosis” in skin cancer screening. Accordingly, this study adopts Swin-T as the basic visual backbone network for subsequent multimodal fusion.

To systematically verify the effectiveness of the proposed Meta-Gate gating mechanism, LR-HDF low-rank hyper-fusion module and multiple training strategies respectively, this study designs multiple groups of ablation control experiments on the independent test set and conducts quantitative comparison with conventional fusion methods. The detailed performance metrics of all compared models are presented in Table 2.

**Table 2:** Performance comparison of each model.

Model configuration	Params (M)	FLOPs (G)	FPS (RTX5070)	CPU FPS	Global AUC	pAUC
Baseline (pure visual Swin-T)	27.50	8.74	112.3	20.11	0.9071	0.8124
Simple Concat (feature concat + meta)	27.72	8.74	114.0	20.31	0.9156	0.8320
Gating Net (only gating introduced)	27.79	8.74	116.6	20.37	0.9190	0.8280
HyperNet (LR-HDF Only)	28.24	8.74	129.0	20.36	0.9117	0.8084
Metadata modulation	27.79	8.74	127.1	20.81	0.9112	0.7035
Cross-attention transformer	27.80	8.74	119.1	19.87	0.7465	0.3319
Bilinear pooling	40.08	8.77	116.7	19.64	0.9149	0.7196
GLHF-Net (*r* = 16, without strong regularization)	28.84	8.74	117.9	20.05	0.9132	0.8282
GLHF-Net (regularized) [ours, *r* = 4]	28.04	8.74	121.1	20.35	0.9200	0.8405

Table 2 presents the experimental results of the ablation study on each functional module. To eliminate the interference caused by differences in computational complexity, all experimental groups are controlled to have a comparable floating-point operation volume (approximately 8.74 G). The pure-visual Swin-T baseline achieves a pAUC of only 0.8124. The direct feature concatenation scheme (Simple Concat), constrained by the semantic gap between heterogeneous features, only improves the pAUC to 0.8320, verifying the inherent limitation of the traditional early fusion strategy. When the Meta-Gate module is introduced alone, the model increases the AUC to 0.9190 with a marginal parameter overhead of 0.29 M, confirming that metadata-driven channel screening can effectively suppress cross-modal negative transfer. In contrast, deploying the high-rank LR-HDF (*r* = 16) alone yields a pAUC even lower than that of the baseline, which is attributed to fusion noise arising from the absence of pre-feature purification. Building upon this insight, GLHF-Net (*r* = 16), which integrates both the Meta-Gate and LR-HDF modules but omits strong regularization, achieves a modest performance gain, yet parameter redundancy still caps its generalization upper bound. The complete GLHF-Net scheme proposed in this study, adopting the *r* = 4 low-rank constraint combined with joint regularization, attains the optimal performance: with 28.04 M parameters and an inference speed of 121.1 FPS, it achieves a global AUC of 0.9200 and a pAUC of 0.8405.

To comprehensively evaluate our approach against recent state-of-the-art multimodal paradigms, we implemented three additional robust baselines: Metadata-Conditioned Modulation, Multimodal Bilinear Pooling, and a Cross-Attention Transformer. As shown in Table 2, while Metadata-Conditioned Modulation is computationally fast (20.81 FPS on CPU), its simple linear scaling fails to capture deep cross-modal semantics, resulting in a substantially lower pAUC (0.7035). Conversely, deep interaction strategies like Bilinear Pooling incur significant computational overhead (40.08 M parameters), leading to mild overfitting (pAUC 0.7196). Notably, the Cross-Attention Transformer, despite extensive hyperparameter re-optimization (learning rate reduced to 1 × 10^−5^ and dropout increased to 0.6) to prevent optimization collapse, still exhibited suboptimal performance (AUC 0.7465, pAUC 0.3319). This reveals the inherent vulnerability of data-hungry full-rank attention mechanisms when applied to small, imbalanced medical datasets; without explicit capacity constraints, they are highly susceptible to severe cross-modal overfitting.

Furthermore, to address the feasibility of real-world clinical deployment—particularly in primary healthcare settings lacking GPU infrastructure—we benchmarked the CPU inference efficiency of all models. Table 2 demonstrates that GLHF-Net (*r* = 4) achieves a real-time CPU processing speed of 20.35 FPS, which is virtually identical to the pure-visual Swin-T baseline (20.11 FPS). By maintaining robust real-time CPU efficiency while securing the highest discriminative accuracy (pAUC 0.8405), GLHF-Net achieves an optimal Pareto efficiency, proving its viability for deployment on portable, low-power screening terminals.

Figure 5 presents the training convergence behavior of all nine models under the unified early stopping constraint. Subfigure (a) illustrates the training loss curve plotted on a logarithmic scale, and subfigure (b) demonstrates the variation trend of AUC values on the validation set. Notably, the Cross-Attention model struggles to converge smoothly and exhibits significant oscillation, reflecting the severe difficulty of optimizing heavy full-rank attention modules on limited medical data. Meanwhile, our regularized GLHF-Net achieves the most stable validation AUC progression, effectively solving the overfitting problem. The joint regularization strategy and low-rank structural constraint produce a powerful synergistic effect, enabling our model to consistently maintain the lowest training loss while securing the highest validation AUC bound.

**Figure 5:**
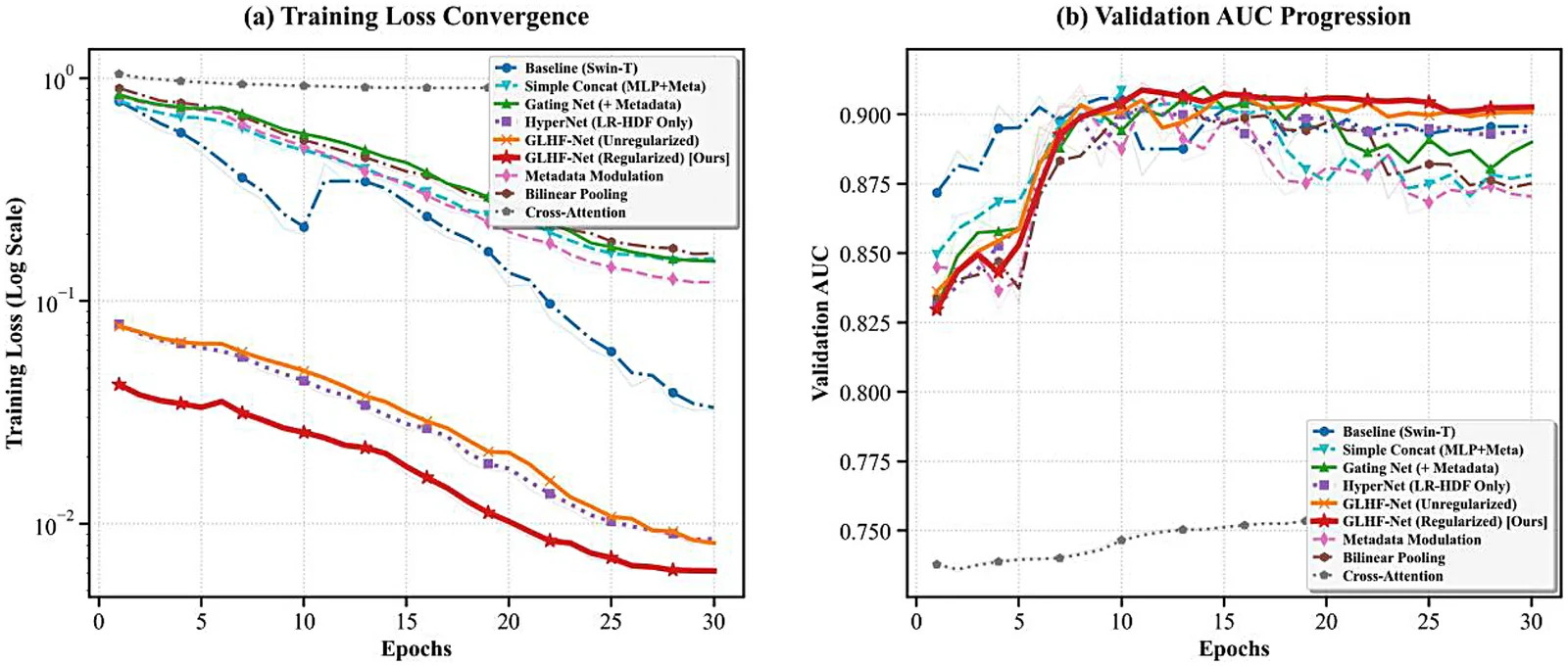
Training process curves of each ablation model: **(a)** training loss decline curve; **(b)** validation set global AUC evolution trend.

Furthermore, to comprehensively evaluate the robustness of the model under different classification thresholds, this study draws the Receiver Operating Characteristic (ROC) curve on the independent test set (see Figure 6).

**Figure 6:**
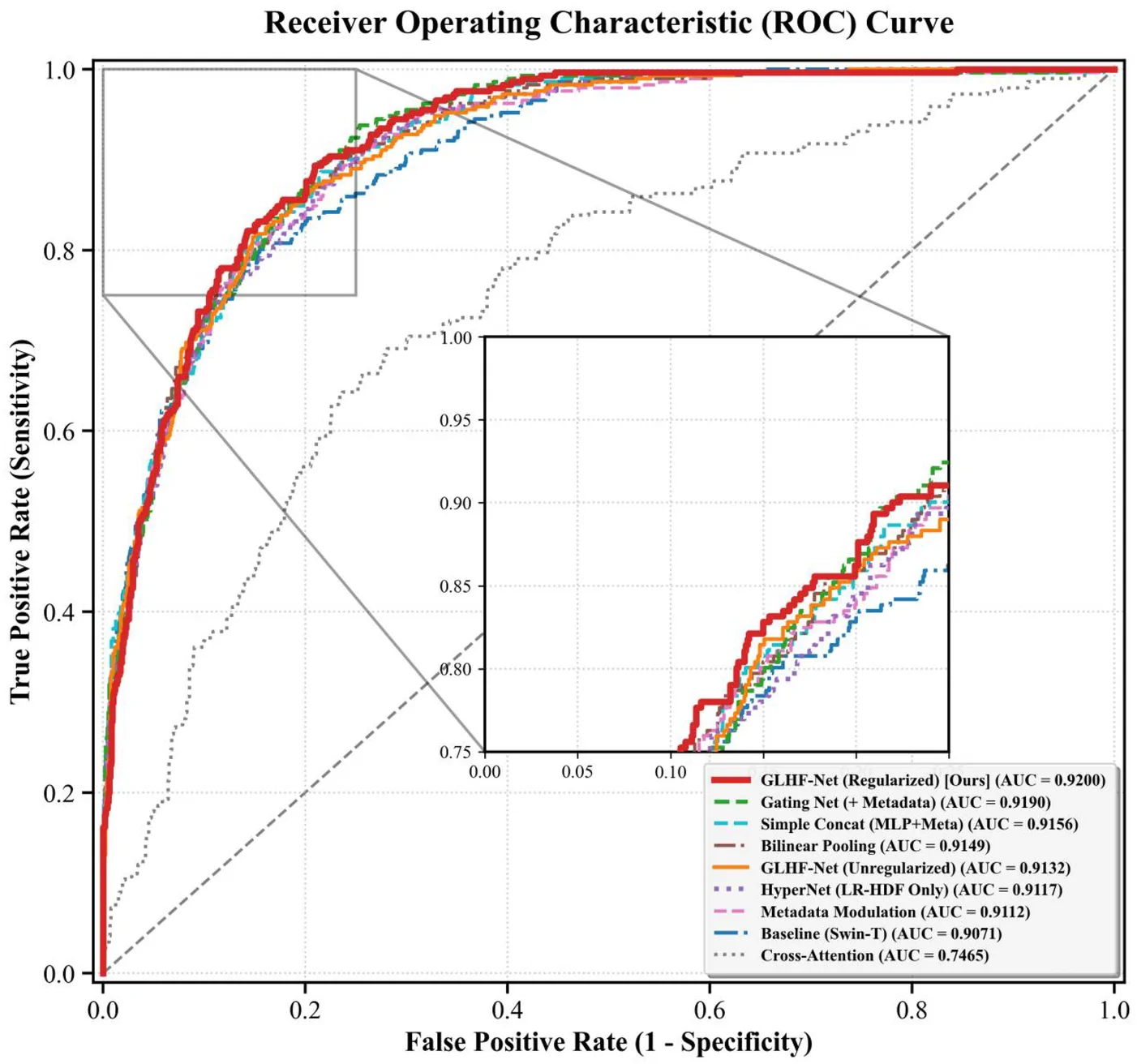
Receiver operating characteristic (ROC) curve and local magnification on the independent test set.

As illustrated in Figure 6, the Receiver Operating Characteristic (ROC) curves for all nine models are systematically compared. Within the clinically critical high-sensitivity interval (where the True Positive Rate ranges from 0.75 to 1.0, shown in the local magnification), the curve for GLHF-Net (Regularized) completely envelops all baseline methods, including the newly added Bilinear Pooling and Metadata Modulation paradigms. This demonstrates that for any pre-defined false positive rate level, the GLHF-Net model obtains a higher true positive rate than all baseline models, which directly reflects the significant improvement of its partial Area Under the Curve (pAUC) performance. The experimental result indicates that, while satisfying the clinical requirement of a low missed diagnosis rate, the proposed model in this study generates the fewest false positive predictions, thereby minimizing the occurrence of unnecessary invasive biopsy procedures to the greatest extent, and exhibits superior practical value for clinical disease screening.

### 4.3 Sensitivity analysis of low-rank hyperparameter r

In the LR-HDF model, the rank *r* determines the parameter capacity of the dynamic weight matrix. Considering the dimensions of the input features (768 dimensions) and output features (256 dimensions), this study selects candidate values r∈{2,4,8,16}, which covers the range from extreme feature compression to moderate parameter redundancy, to conduct sensitivity analysis. As illustrated in Table 3, when *r* = 16 or *r* = 8, the model tends to overfit due to excessive parameter capacity; when *r* is compressed to 2 a bottleneck occurs in information transmission. When and only when r=4, the model achieves the optimal balance between prediction accuracy and anti-overfitting capability.

**Table 3:** Comparison of different ranks *r*.

Rank (r)	Best Val AUC	Best Val pAUC	Status analysis
2	0.9091	0.8042	Excessive compression leads to information bottlenecks
4	0.9200	0.8405	The optimal balance between parameter size and generalization capability
8	0.9039	0.8033	Mild overfitting
16	0.9074	0.8138	Redundant parameters lead to overfitting

### 4.4 Clinical diagnostic efficacy and confusion matrix analysis

In the early clinical screening of malignant tumors, the diagnostic system is required to strike a balance between high sensitivity and reasonable specificity. In this study, the optimal classification threshold was determined via the Youden index (calculated as sensitivity + specificity − 1) using the validation set, and the resulting optimal threshold was 0.402. After determining the threshold, it was directly applied to the independent test set to calculate all clinical diagnostic indicators, which effectively avoids indicator inflation caused by test set data leakage. As presented in Table 4, the sensitivity of the GLHF-Net (Regularized, *r* = 4) model reaches 0.8935. More notably, the Negative Predictive Value (NPV) of this model achieves 0.9682. This result verifies that as an auxiliary pre-diagnosis tool, GLHF-Net can exclude low-risk lesions with extremely high confidence, thereby substantially reducing unnecessary invasive biopsies and alleviating the burden of the medical system.

**Table 4:** Clinical diagnostic efficacy indicators on the independent test set.

Clinical metrics	Value	Clinical significance explanation
Accuracy	0.8109	Global Classification Accuracy
Sensitivity/Recall	0.8935	Highly effective in preventing missed diagnosis of malignant lesions
Specificity	0.7908	Enhance the ability to identify benign lesions and prevent misdiagnosis
PPV	0.5098	Indicates high-risk status and guides biopsy performance
NPV	0.9682	High-confidence exclusion of malignancy to reduce unnecessary surgeries
*F*1-score	0.6492	Comprehensive performance evaluation under severe imbalance conditions

### 4.5 Interpretability analysis

To validate the inherent feature purification and noise suppression performance of the proposed model, we eliminated the hard ROI masking step from the data preprocessing stage in this specific visualization experiment. This experimental design rules out the interference introduced by manual preprocessing operations, thereby allowing an unbiased assessment of the model's endogenous noise reduction ability and lesion region-focused attention mechanism. The corresponding visualization results are shown in Figure 7.

**Figure 7:**
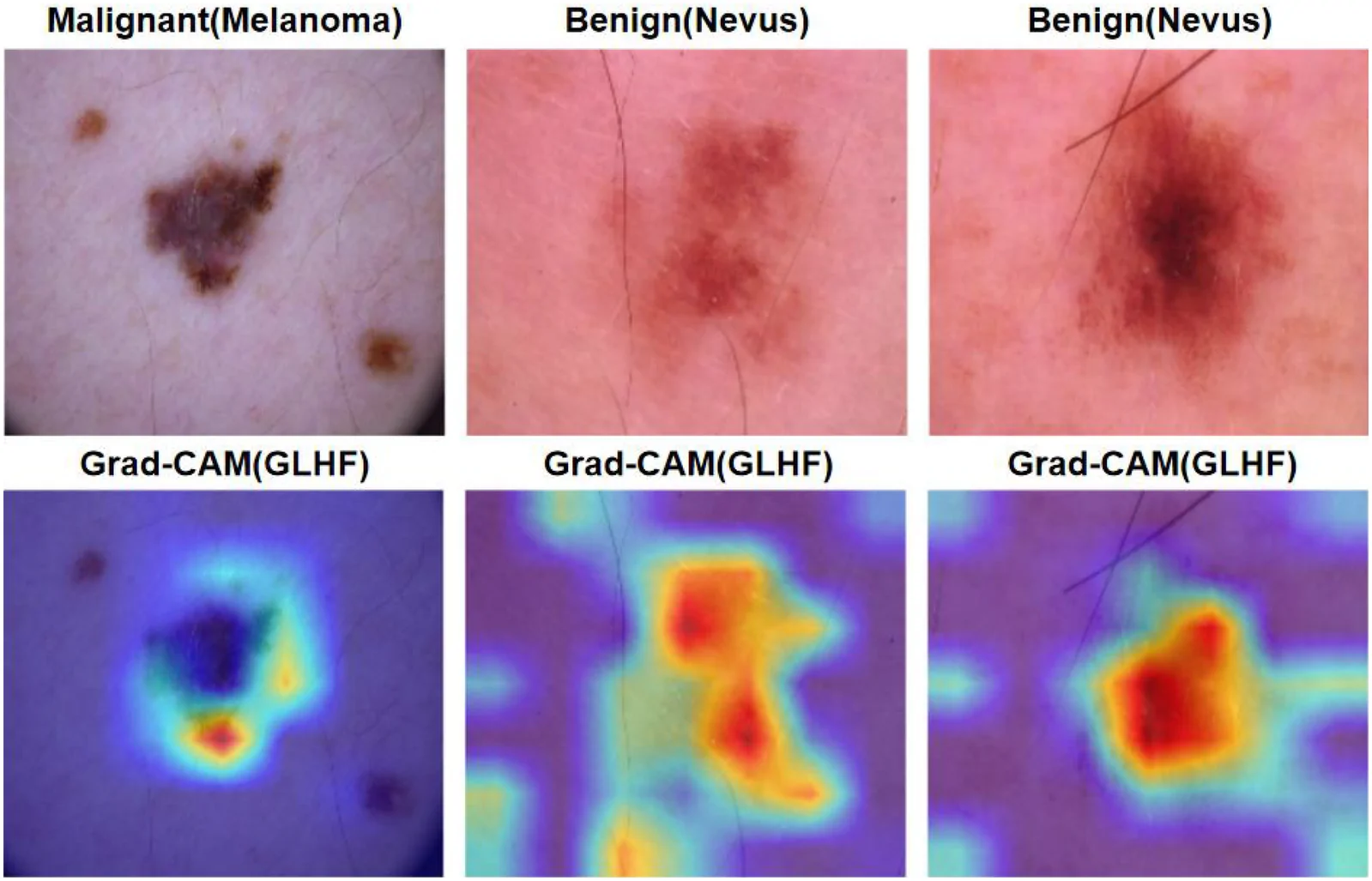
Grad-CAM heatmaps.

In Figure 7, the first row displays three original dermatoscopic images collected from different samples, and the second row shows the corresponding Grad-CAM heatmaps output by the GLHF-Net model. In these heatmaps, regions with colors approaching red correspond to areas that received higher attention from the model. The results indicate that, without the assistance of any hard ROI mask, the proposed GLHF-Net model (second row of Figure 7) is able to substantially suppress complex hair-related noise and bubble artifacts within its high-sensitivity activation centers. The model autonomously realizes channel-level noise reduction, and can accurately and stably focus attention on the core pathological regions of melanoma and dysplastic nevus, with activation areas highly consistent with the key pathological features defined by the classic ABCD diagnostic criteria, including the pigment network and irregular morphological margins.

To quantitatively verify this robustness against noisy and unmasked inputs frequently encountered in real clinical practice, we additionally evaluated both GLHF-Net and the pure-visual Swin-T baseline on raw dermoscopic images (without the hard ROI mask) using the identical independent test set. As presented in Table 5, given the absence of pixel-level pathological segmentation masks, this unmasked evaluation serves as a compelling quantitative proxy for the model's interpretability. Although the hard ROI mask yields the best absolute performance for GLHF-Net (global AUC = 0.9200, pAUC = 0.8405), the model retains a strong diagnostic capability on raw images (global AUC = 0.9179, pAUC = 0.8403). Conversely, on the same raw images, the pure-visual Swin-T baseline achieves a global AUC of 0.9269 but a significantly lower pAUC of 0.7494. Although the baseline exhibits a slightly higher global AUC by exploiting background correlations, GLHF-Net delivers a substantially higher pAUC under the high-sensitivity regime (TPR ≥ 0.8) that is most relevant to clinical screening. This quantitative resilience corroborates the qualitative Grad-CAM findings, confirming that the network inherently focuses on core pathological features rather than blindly memorizing background artifacts.

**Table 5:** Comparison of GLHF-Net performance with and without hard ROI masking on the independent test set.

Setting	Global AUC	pAUC
Pure-visual Swin-T (with mask)	0.9071	0.8124
Pure-visual Swin-T (without mask)	0.9269	0.7494
With hard ROI mask	0.9200	0.8405
Without hard ROI mask	0.9179	0.8403

### 4.6 External validation on the ISIC 2019 dataset

To rigorously validate the cross-center generalization ability of the proposed method and directly address whether the hyperparameter configuration optimized on the source domain is transferable, GLHF-Net was evaluated on the ISIC 2019 skin lesion classification challenge dataset (23).

We first tested the model under the exact same settings used for the HAM10000 analysis, strictly inheriting the low-rank constraint of *r* = 4. As shown in Table 6, GLHF-Net (*r* = 4) experiences a performance drop compared to the pure-visual Swin-T baseline. This explicitly reveals the vulnerability of applying source-optimized capacity directly to out-of-distribution (OOD) data, where domain shift causes the redundantly parameterized fusion module to introduce heterogeneous noise.

**Table 6:** Performance comparison on the external ISIC 2019 dataset.

Model configuration	Global AUC	pAUC	Accuracy
Pure-visual Swin-T (baseline)	0.9612	0.8400	0.8967
GLHF-Net (ours, *r* = 2)	0.9576	0.8393	0.9171
GLHF-Net (ours, *r* = 4)	0.9533	0.8084	0.8859

However, when adapting to the stricter information bottleneck for external data (*r* = 2), the model not only recovers its discriminative power (Global AUC 0.9576, pAUC 0.8393) but, most importantly, significantly outperforms the pure-visual baseline in diagnostic Accuracy (0.9171 vs. 0.8967). This highlights a crucial clinical advantage of our architecture: while baseline visual features might maintain high generalized probabilistic ranking (AUC) on unseen data, the properly constrained multimodal fusion (*r* = 2) effectively recalibrates hard boundary cases. By integrating clinical metadata under an optimal low-rank constraint, the model corrects visual ambiguities and leads to more accurate, definitive clinical decisions.

To systematically investigate this phenomenon and verify the optimal rank hyperparameter for external datasets, a comprehensive rank sensitivity analysis (r ∈ {2, 4, 8, 16}) was conducted on the ISIC 2019 dataset using the identical protocol. The results are detailed in Table 7.

**Table 7:** Rank sensitivity analysis on the external ISIC 2019 dataset.

Rank (*r*)	Best Val AUC	Best Val pAUC	Status analysis
2	0.9576	0.8393	Optimal setting for external generalization, extracting domain-invariant features via a strict information bottleneck.
4	0.9533	0.8084	Slight performance degradation due to marginally increased parameter capacity.
8	0.9515	0.7937	Further decline in out-of-distribution discriminative ability.
16	0.9501	0.7854	Suboptimal performance with the lowest pAUC, indicating overfitting to the source domain.

The external validation results in Table 7 yield an insightful observation regarding the model's behavior on out-of-distribution (OOD) data. Interestingly, we observe a strict monotonic decrease in both Global AUC (from 0.9576 to 0.9501) and the clinically critical pAUC (from 0.8393 to 0.7854) as the rank hyperparameter increases from *r* = 2 to *r* = 16. While *r* = 4 was the optimal configuration on the source HAM10000 dataset, the stricter constraint of *r* = 2 emerges as the absolute optimal setting for the external ISIC 2019 dataset.

This discrepancy is theoretically well-founded. When transferring to unseen data from different clinical centers and acquisition devices, domain shift inevitably occurs. A larger parameter capacity (e.g., *r* = 8 or 16) allows the fusion module to memorize domain-specific artifacts and redundant noise inherent to the source dataset, resulting in suboptimal external generalization. Conversely, imposing a tighter low-rank information bottleneck (*r* = 2) forces the LR-HDF module to discard domain-specific bias and strictly retain only the most robust, domain-invariant pathological features. This finding not only validates the strong anti-overfitting ability of the proposed framework but also highlights the architectural superiority of GLHF-Net: by simply adjusting the low-rank factor r, the model can flexibly control its capacity to achieve optimal cross-center robustness in diverse clinical deployment scenarios.

### 4.7 Model calibration and reliability

For an AI-assisted diagnostic system to be safely adopted in clinical practice, it must not only achieve high classification accuracy but also output well-calibrated probabilities. A well-calibrated model ensures that its predicted confidence precisely reflects the true likelihood of malignancy, providing reliable risk stratification for dermatologists.

To rigorously assess this, we evaluated the probabilistic calibration of GLHF-Net using the Expected Calibration Error (ECE) and a Reliability Diagram (Figure 8B). The reliability curve closely tracks the ideal diagonal (perfect calibration), indicating that the model neither over-confidently predicts benign lesions nor underestimates the risk of malignant ones. The model achieves an Expected Calibration Error (ECE) of 0.1518. Although this ECE value indicates a moderate overestimation of malignancy risk, this is an intentional and clinically favorable trade-off. Driven by the Focal Loss optimization (which heavily penalizes minority malignant classes) and the clinically tuned threshold, the model strictly penalizes false negatives to prioritize high sensitivity. This ensures patient safety in triage scenarios by aggressively flagging potential malignancies, achieving an exceptionally high NPV (0.9682) even at the expense of marginal calibration drift. Thus, the output probabilities generated by the LR-HDF fusion module are highly trustworthy for conservative clinical decision-making.

**Figure 8:**
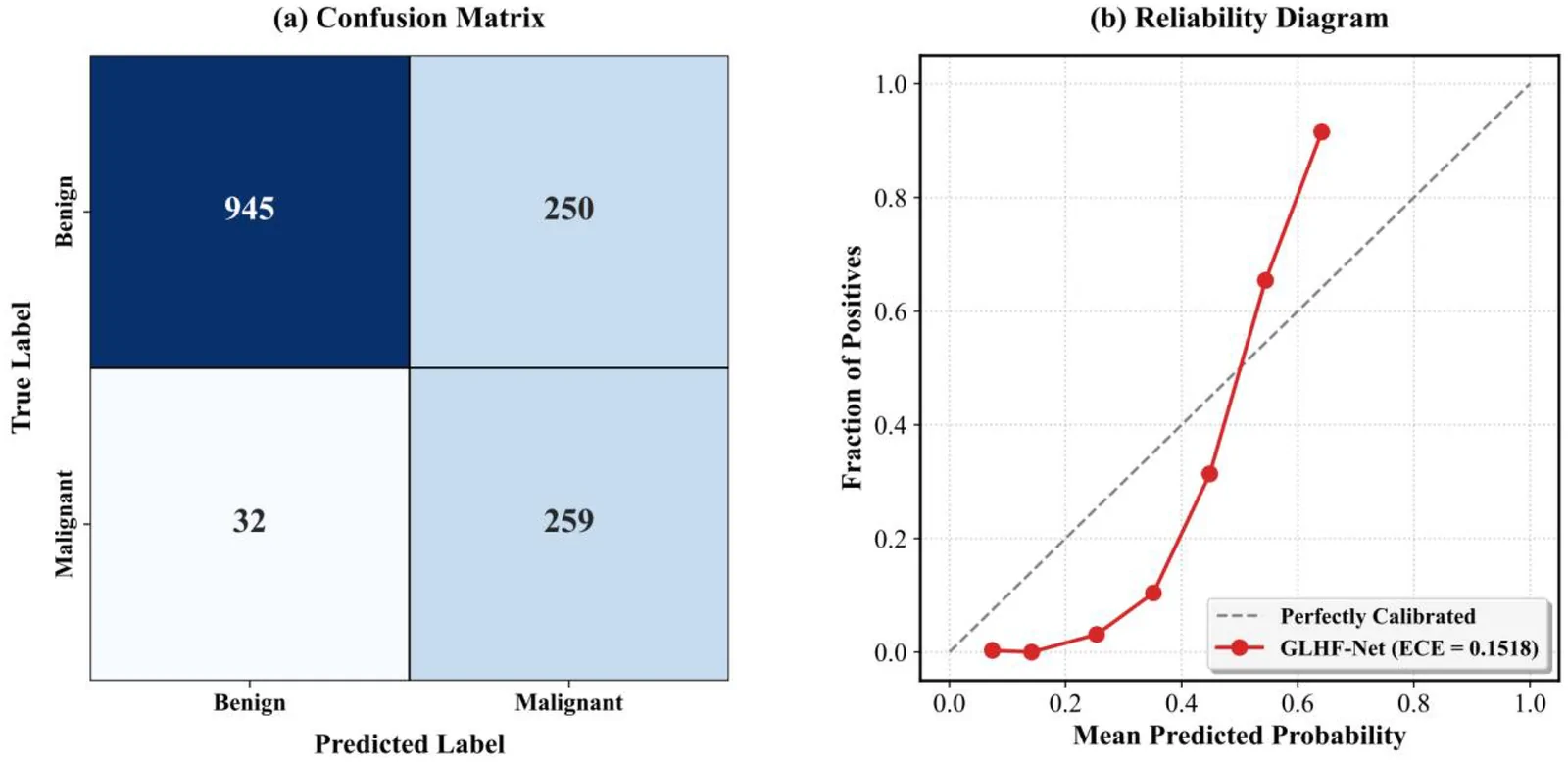
Expected calibration error (ECE) and model reliability evaluation. (**a**) Confusion matrix. (**b**) Reliability diagram.

## 5 Discussion

### 5.1 Core innovations and performance superiorities of the proposed model

Aiming at three core challenges, namely cross-modal negative transfer, overfitting under small sample conditions and insufficient model lightweighting in multimodal skin lesion diagnosis, the GLHF-Net model proposed in this study achieves performance improvement via a two-stage progressive fusion architecture. First, the Meta-Gate channel-wise gating mechanism leverages clinical metadata to guide the purification of visual features, which effectively eliminates heterogeneous modal interference. The introduction of this module alone improves the partial Area Under the Curve (pAUC) of the pure visual Swin-T baseline by 1.92%. Second, the LR-HDF low-rank hyper-fusion module imposes an extremely low-rank constraint (*r* = 4), reducing fusion-layer parameters by 97.9% while preserving patient-specific dynamic fusion capability. This structural design effectively suppresses small-sample overfitting. Third, the joint optimization anti-overfitting framework further optimizes category distribution balance and model convergence stability, guaranteeing the stable performance of the proposed model on independent test sets. Additionally, visualization results based on Grad-CAM validate that the model's decision-making process focuses on the core pathological regions of lesions, which is consistent with clinical diagnosis and treatment logic and improves the credibility of the model.

### 5.2 Clinical significance and application value of experimental results

On the HAM10000 dataset, the GLHF-Net model obtains the optimal performance with a global AUC of 0.9200 and a pAUC of 0.8405 in the high-sensitivity interval, representing a 3.46% pAUC improvement over the pure visual baseline. Under the optimal threshold determined by maximizing the Youden's index, the sensitivity of the model reaches 0.8935 (corresponding to a missed diagnosis rate of 10.65%), which satisfies the core requirement of “missed diagnosis prevention” in early skin cancer screening. Meanwhile, its negative predictive value reaches 0.9682, enabling the exclusion of low-risk lesions with extremely high confidence and significantly reducing unnecessary invasive biopsies. In addition, the proposed model only contains 28.04 M parameters and achieves an inference frame rate of 121.1 FPS. It can be deployed on grassroots portable screening devices without dedicated high-end computing resources, demonstrating favorable clinical translation potential. Moreover, the strict patient ID-based data partitioning strategy adopted in this study avoids data leakage and effectively verifies the actual generalization ability of the model.

### 5.3 Research limitations

This study presents certain limitations that warrant future investigation: First, although external validation has been performed on the ISIC 2019 dataset, validation on more heterogeneous data from diverse imaging devices and clinical settings remains to be conducted. Second, while the binary classification (benign vs. malignant) strategically aligns with the primary clinical objective of biopsy triage, it simplifies the original 7-class diagnostic task of the dataset. Finally, the proposed fusion framework exhibits an inherent dependency on metadata quality. While the unknown-category placeholder mechanism handles missing values gracefully, extreme real-world scenarios with systematically corrupted or completely unavailable clinical records may still constrain the fusion module's auxiliary diagnostic potential.For future work, we plan to expand the dimension of multimodal fusion by integrating clinical information including medical history and pathological data, so as to improve the classification accuracy of difficult-to-distinguish lesions. In addition, we will advance the lightweight deployment of the model and develop portable auxiliary skin cancer screening terminals suitable for primary medical institutions.

## 6 Conclusions

### 6.1 Research conclusions

To address the core limitations of existing multimodal intelligent diagnostic models for skin lesions, this study proposes a lightweight Gated Low-Rank Hyper-Fusion Network (GLHF-Net) and establishes a two-stage fusion framework with the configuration of “purification prior to fusion”. Experimental results indicate that the proposed Meta-Gate mechanism can mitigate cross-modal negative transfer, and the LR-HDF module achieves an optimal Pareto efficiency between model lightweighting and anti-overfitting performance. On the independent test set, the proposed model obtains excellent performance with a global AUC of 0.9200, a pAUC of 0.8405, a sensitivity of 0.8935, and a negative predictive value of 0.9682, while maintaining an ultra-high inference efficiency of 121.1 FPS on GPU and 20.35 FPS on CPU. Furthermore, external validation with retraining on the independent ISIC 2019 multi-center dataset confirms its robust out-of-distribution generalization. Notably, by adaptively tightening the low-rank information bottleneck (e.g., *r* = 2), GLHF-Net effectively filters out domain-specific artifacts, maintaining high diagnostic accuracy across diverse clinical centers.

By synergizing lightweight model design with interpretable decision-making, GLHF-Net narrows the gap between sophisticated deep learning research and practical clinical deployment. This study not only promotes methodological development in the field of multimodal fusion, but also provides a feasible solution for large-scale non-invasive skin cancer screening, which is of particular application value for primary healthcare scenarios.

### 6.2 Future prospects

Future research will be carried out from three main aspects: first, constructing a multi-center heterogeneous dataset to systematically validate the out-of-domain generalization performance of the proposed model; second, expanding the dimension of multimodal fusion by integrating clinical information including medical history and pathological data, so as to improve the classification accuracy of difficult-to-distinguish lesions; third, advancing the lightweight deployment of the model and developing portable auxiliary skin cancer screening terminals suitable for primary medical institutions.

## Data Availability

The original contributions presented in the study are included in the article, further inquiries can be directed to the corresponding author.
